# Unilateral Facial Swelling in a Sickle Cell Patient

**DOI:** 10.7759/cureus.66693

**Published:** 2024-08-12

**Authors:** Rohan Akhouri, Ali Fowler, Colton T Schwarz, Lina Patel

**Affiliations:** 1 Pediatric Emergency Medicine, Children's Mercy Hospital, Kansas City, USA; 2 Pediatrics, Children's Mercy Hospital, Kansas City, USA

**Keywords:** acute soft head syndrome, hair-on-end appearance, facial swelling, pediatric emergency department, sickle cell complications

## Abstract

Acute soft head syndrome (ASHS) is a rare complication of sickle cell disease that often requires a high index of suspicion and is often a diagnosis of exclusion. We present the case of an 18-year-old male with sickle cell disease in the United States who developed acute soft head syndrome without known traumatic injury. The goal of this case presentation is to provide awareness and education regarding a rare complication of sickle cell disease and recommended management for the associated symptoms.

## Introduction

Sickle cell disease affects approximately 1 in 500 African Americans [1]. Acute soft head syndrome (ASHS) is a rare complication of sickle cell disease that often requires a high index of suspicion. Although guidelines for other more common complications of sickle cell disease exist, none exist for ASHS. The literature is primarily composed of individual case reports for this phenomenon. Acute soft head syndrome has been reported more commonly among adolescent males with a history of hemoglobin SS (HbSS) disease and often is in association with an acute vaso-occlusive crisis [2-12]. It is not clear why this population may be more often affected but theories proposed include bone marrow hyperplasia, chronic bony changes from increased hematopoiesis, and possible physiologic changes in males during puberty and adolescent development [3]. 

## Case presentation

An 18-year-old male with a history of hemoglobin SS disease (HbSS), asthma, and choledocholithiasis presents to a pediatric emergency department in the United States for evaluation of acute left-sided facial and scalp swelling. The patient had an endoscopic retrograde cholangiopancreatography (ERCP)** **10 days prior and was in his normal state of health until 4 days prior to presentation when he described a tension headache that improved with ibuprofen and acetaminophen. The following day, he described bilateral thigh pain that also resolved with over-the-counter analgesics. He noted swelling along the left side of his face and scalp approximately 18 hours prior to presentation that has progressively increased in size and tenderness. The patient reports his last vaso-occlusive pain crisis was many years ago, however, the recent thigh discomfort is similar to past vaso-occlusive pain. 

On presentation, the patient reported a headache that was described as a “pressure” and rated 2/10 in severity. There was no reported history of head trauma, insect bites/stings, or other injuries to the affected area. He denied any pain with swallowing or eye movements. No fevers, chills, recent viral respiratory symptoms, or illnesses. No visual disturbances. He had no known history of allergies. 

**Figure 1:**
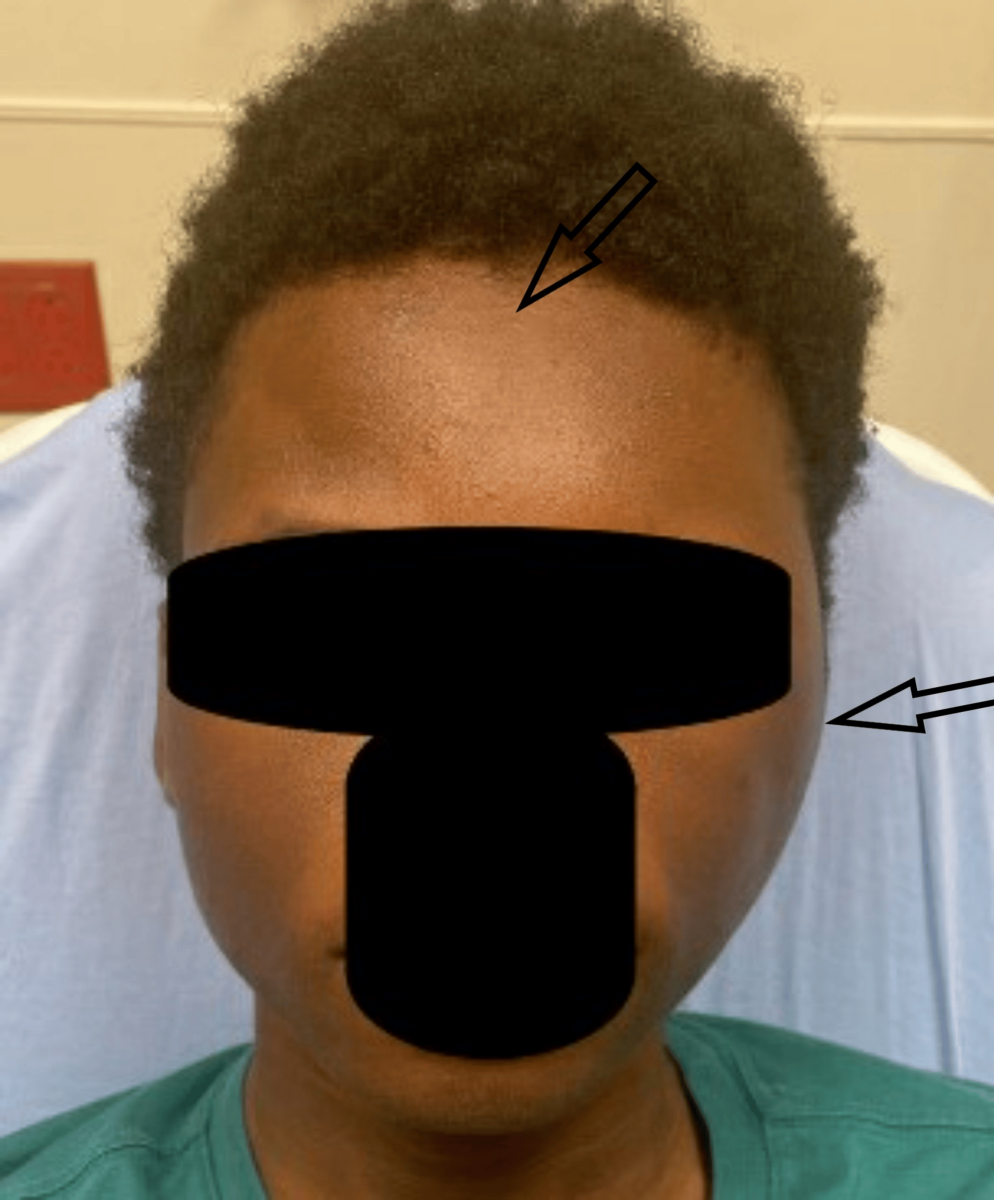
Facial swelling at initial presentation

Initial vital signs were appropriate for his age - temp 37.0C, HR 94 bpm, RR 23/min, BP 124/58, SpO2 98%. On exam, the patient was well-appearing with diffuse, well-demarcated soft tissue edema along the left face extending superiorly from the left maxilla to the left parietal scalp and slightly medially across the midline of the forehead (Figure 1). No erythema, fluctuance, induration, warmth, or overlying skin changes were noted. The patient had mild tenderness along the area of swelling, most significantly along the left parietal scalp. Ears, oropharynx, and nares exam were normal. There was no cervical lymphadenopathy. The remainder of the physical exam was normal. 

**Figure 2:**
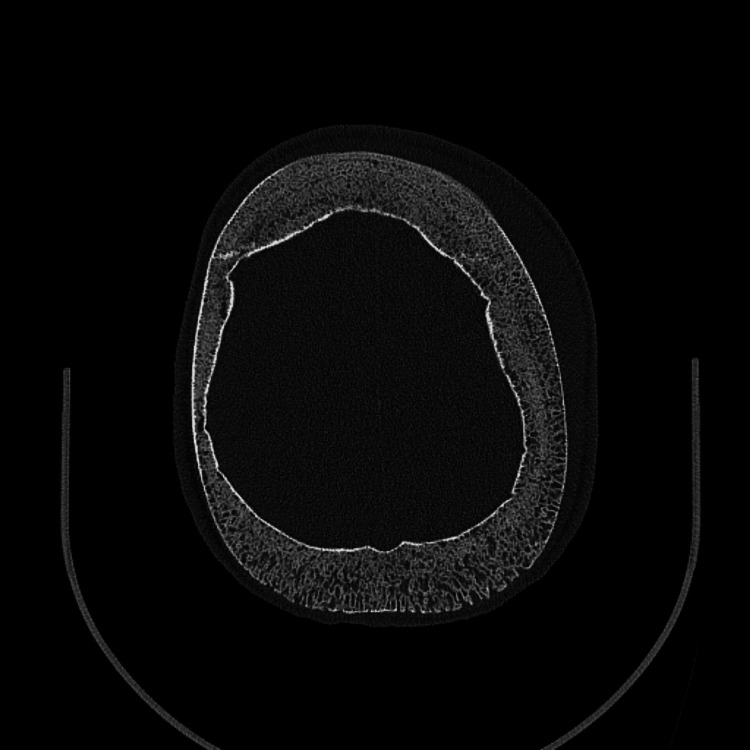
Hair-on-end appearance on head and maxillofacial CT

**Figure 3:**
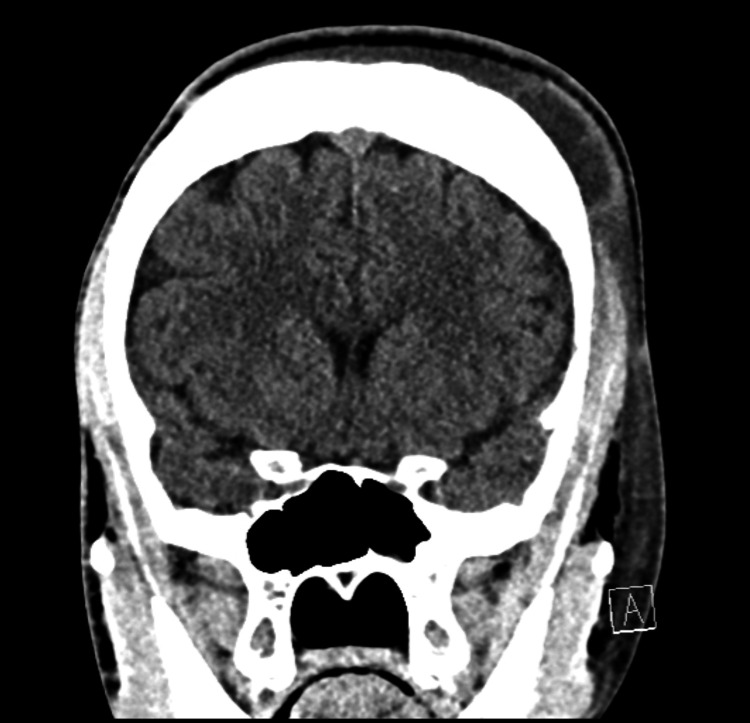
Soft tissue swelling noted on head and maxillofacial CT

Labs were obtained that revealed a leukocytosis (17.86 x10(3)/mcL) with elevated absolute neutrophil count (11.61 x10(3)/mcL), elevated C-reactive protein (20.2 mg/dL), microcytic anemia (Hgb 10.0 gm/dL, MCV 71.3 fL) which was near his baseline (Hgb 10.2 gm/dL), elevated absolute reticulocyte count (0.31 x10(6)/mcL), and a normal basic metabolic panel. Intranasal fentanyl was provided for pain with good relief. Head and maxillofacial CT without contrast was obtained. This imaging showed generalized thickening of the calvarium with a “hair on end” configuration (Figure 2), no definitive source of focal osteoporosis or erosion to account for the soft tissue swelling, nonspecific left-sided frontoparietal scalp edema with possible partial organization of the fluid (Figure 3).

The patient was admitted to the inpatient hematology service and was initially treated with intravenous fluids and ibuprofen as needed for pain. Shortly after admission, he developed a fever of 39.5°C and was treated empirically with ceftriaxone and vancomycin. This regimen was chosen to broadly cover skin/soft tissue infections, meningitis, and other intracranial infections, as there were no published guidelines for antimicrobial treatment of acute soft head syndrome.

**Figure 4:**
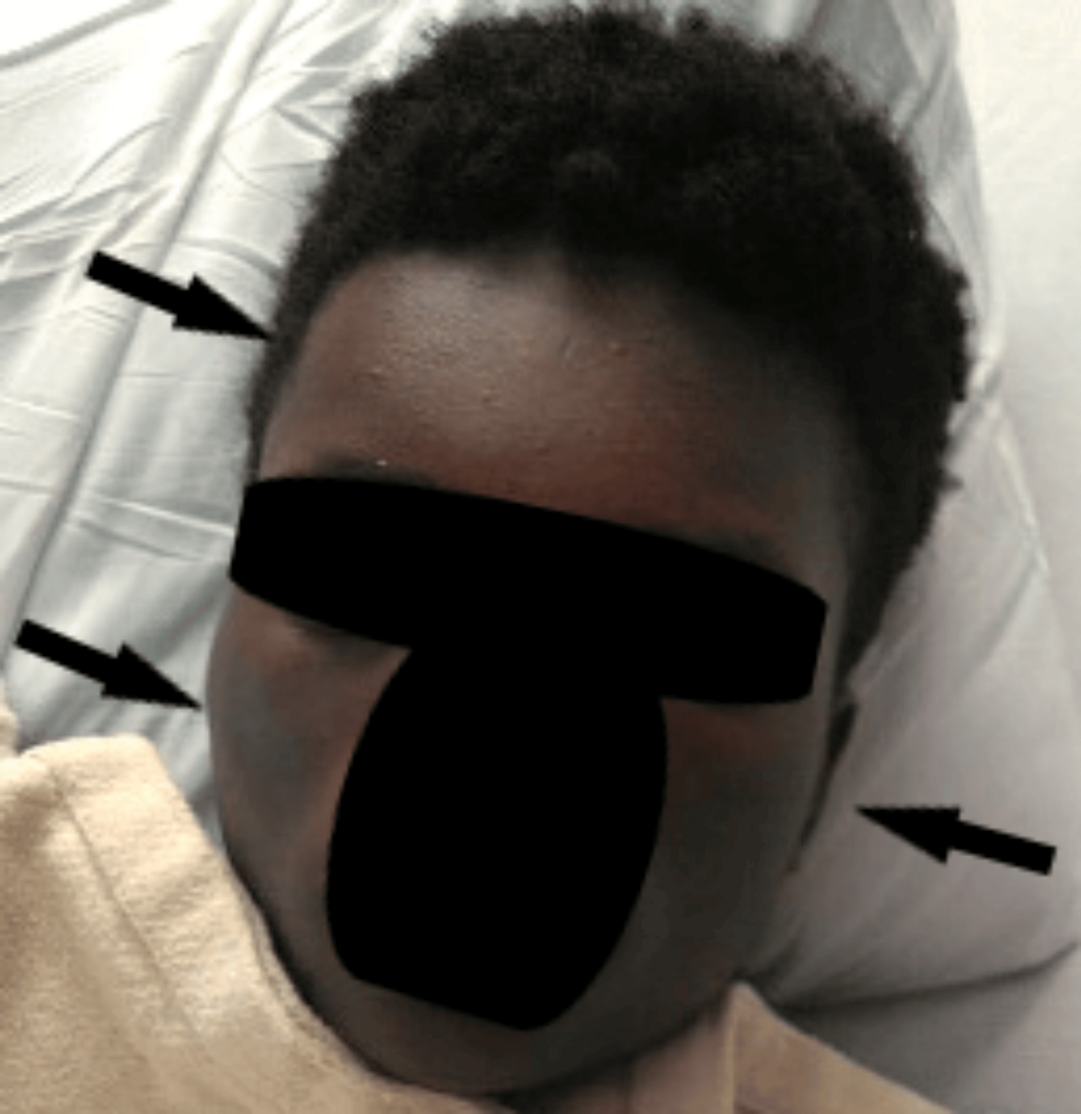
Worsened facial swelling on day 2

**Figure 5:**
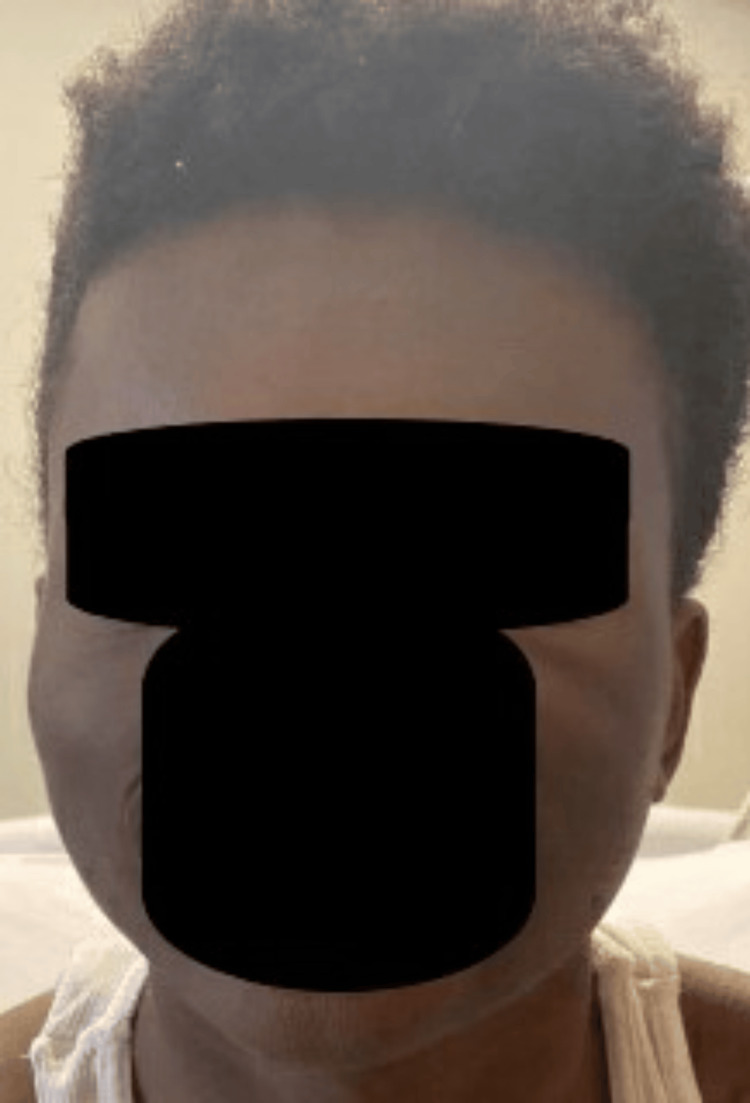
Improvement and resolution of facial swelling

The following morning, his facial swelling progressed to include the left mandible and neck (Figure 4). The infectious disease service was consulted and recommended that his antimicrobial coverage be narrowed to cefazolin for targeted treatment of gram-positive skin and soft tissue infections, as the patient had no methicillin-resistant *Staphylococcus aureus *(MRSA) risk factors or evidence of intracranial infection. He had no further fevers, and his blood culture obtained on presentation showed no growth. Repeat labs demonstrated down-trending leukocytosis (15.3 x10(3)/mcL) and absolute neutrophil count​​​​​​​ (ANC)(10.11 x10(3)/mcL). His facial swelling improved (Figure 5), and he was discharged two days after presentation with a discharge plan to complete 5 days of oral cephalexin.

## Discussion

Although the pathophysiology for ASHS is not well understood and the explanations are likely multifactorial, several proposed theories have been suggested. There may often be a preceding or current vaso-occlusive pain crisis upon presentation leading to infarctions of the calvarium, or recurrent micro-infarctions that lead to disruption of the inner and/or outer layer of the skull and subsequent extravasation of blood into the epidural, subdural, or subgaleal space [4,8]. Other theories also include expansion of the intramedullary tissue from increased hematopoiesis due to chronic anemia which also can result in cortical disruption. Local hypoxemic stress can lead to the development of new and fragile blood vessels which may be more predisposed to rupture [3,6,7]. 

Acute soft head syndrome is a diagnosis of exclusion in patients with sickle cell disease and a high index of suspicion for their isolated symptoms. Based on case reports of ASHS, most patients present with non-traumatic headaches and isolated swelling that may be tender and can expand diffusely if in the subgaleal space [3,4]. Patients may develop fevers, leukocytosis, and associated bone pain. It can be challenging to differentiate osteomyelitis, acute pain crises, and ASHS based on the initial presentation and laboratory findings [4,8]. 

Patients with sickle cell disease presenting in the emergency setting should undergo rapid assessment and treatment for disease-associated crises such as acute chest syndrome, stroke, and splenic sequestration [13]. Common initial laboratory testing includes complete blood count (CBC) with differential, lactate dehydrogenase (LDH), reticulocyte count, basic metabolic panel (BMP), blood type and screen, and blood cultures if concerned for an infectious process [1]. 

In the acute setting for SCD patients with headaches and abnormal exam findings, a CT provides rapid assessment to rule out an intracranial ischemic or hemorrhagic event. A CT can help identify the extent of soft tissue swelling, and help identify the “hair on end” sign representing thickened calvarium due to ineffective erythropoiesis seen in sickle cell disease and other chronic anemic processes [14,15]. An MRI will be more sensitive in identifying any underlying bone infarct in patients with suspected ASHS [4]. The management of ASHS is mostly supportive and similar to the management of other sickle cell-related crises, including IV fluids, pain control, and broad-spectrum antibiotics [8,16,17].

## Conclusions

Our patient represents a rare and benign case of acute soft head syndrome more often seen in adolescent males with HbSS sickle cell disease. The pathophysiology of ASHS is not well understood and is suspected to have a multifactorial etiology. Clinical findings may often demonstrate an acute vaso-occlusive pain crisis, headache, and unilateral soft tissue swelling to the scalp. Often the associated swelling is without a clear etiology, although trauma and infection should be strongly considered. Imaging with non-contrast CT or MRI is helpful to narrow the diagnosis and evaluate for both intracranial and extracranial findings. A non-contrast CT provides rapid assessment for life-threatening intracranial complications from sickle cell-related vaso-occlusive crisis. Acute soft head syndrome should remain high on the differential diagnosis in patients with a history of sickle cell disease presenting with acute scalp swelling with few other preceding symptoms or inciting events. Overall, most cases of ASHS will resolve with conservative management, which includes treating the vaso-occlusive crisis, intravenous fluids, and pain management. 

## References

[1] Sedrak A, Kondamudi NP (2024 Jan-). Sickle cell disease. StatPearls [Internet].

[2] Allen J, Spinks J, Stedman J (2023). Acute soft head syndrome in sickle cell disease. Arch Dis Child.

[3] Drew M, Fladeland D, Sinha R (2024). Acute soft head syndrome in sickle cell anemia: creating a firm approach. Authorea.

[4] Akodu SO, Njokanma OF, Diaku-Akinwumi IN, Ubuane PO, Adediji UO (2014). Acute soft head syndrome in children with sickle cell anaemia in Lagos, Nigeria. Indian J Hematol Blood Transfus.

[5] Zadeh C, Rameh V, Atweh LA (2021). Acute soft head syndrome in a sickle cell disease patient. J Radiol Case Rep.

[6] Taha ZI, Mohammed SE, Essa MEA (2019). Acute soft skull syndrome in an adult male with sickle cell anemia in Sudan: a case report. Explor Res Hypothesis Med.

[7] Magitta NF, Komanya FB, Alphonce BO, Bitesigilwe MD, Sindato EM, Meda JR (2023). Acute soft head syndrome in a teenager with sickle cell anemia: a case report. Clin Case Rep.

[8] Hanafy E, Al Amri S, Alenazi A (2019). Acute soft head syndrome and a mini review of bone and neurologic complications in patients with sickle cell disease. Int J Case Rep.

[9] Mlay IE, Mbwasi RM (2022). Non-traumatic head swellings in a child with a sickle cell disease presented to a tertiary hospital north of Tanzania: a case report. Tanz J Health Res.

[10] Garba N, Ahmadu I, Abubakar M, Asani M, Aliyu I (2022). Acute soft head syndrome in sickle anemia: the first case report in Kano. Med J Dr DY Patil Vidyapeeth.

[11] Westphal K, Respicious Bakalemwa, Groothuis E (2021). Scalp swelling and headache in a 12-year-old boy. Pediatr Rev.

[12] Foula MS, Hassan A, AlQurashi A, Alsaihati A, Sharroufna M (2019). Spontaneous subgaleal hematoma in a patient with sickle cell disease: a case report and literature review. Clin Case Rep.

[13] Kato GJ, Piel FB, Reid CD (2018). Sickle cell disease. Nat Rev Dis Primers.

[14] Martin L, Rackard F (2016). Images in clinical medicine. Hair-on-end sign. N Engl J Med.

[15] Amadi OP, Patel C, Tangella K, Khaliq W (2012). 'Hair-on-end' sign: an important finding in clinical practice. QJM.

[16] AL-Ansari RY, Al Harbi M, Al-Jubair N, Abdalla L (2020). Acute soft head syndrome (subgaleal haematoma) with periorbital oedema as a rare presentation in sickle cell disease. Eur J Case Rep Intern Med.

[17] Darbari DS, Sheehan VA, Ballas SK (2020). The vaso-occlusive pain crisis in sickle cell disease: definition, pathophysiology, and management. Eur J Haematol.

